# Factors Related to Discharge-Oriented Dietary Support for Older Patients with Cancer at a Regional Core Cancer Hospital in Japan: A Cross-Sectional Study

**DOI:** 10.3390/nursrep15110390

**Published:** 2025-11-04

**Authors:** Yoko Kano, Mai Yoshimura, Naomi Sumi

**Affiliations:** 1 Graduate School of Health Sciences, Hokkaido University, Sapporo 060-0812, Japan; kano.yoko.o5@elms.hokudai.ac.jp; 2 Department of Fundamental Nursing, Faculty of Health Sciences, Hokkaido University, Sapporo 060-0812, Japan; mai-yoshimura@hs.hokudai.ac.jp

**Keywords:** neoplasms, aged, nurses, nutrition therapy, cross-sectional study

## Abstract

**Background/Objectives**: Older patients with cancer are predisposed to malnutrition, contributing to adverse postoperative outcomes, high complication rates, and poor prognosis, warranting dietary support from nurses. Practices and factors related to such support remain unexplored. We examined factors associated with discharge-oriented dietary support for older patients with cancer. **Methods**: This cross-sectional study involved registered nurses working in wards and was conducted using a self-report questionnaire between September 2024 and February 2025 at two regional core cancer Japanese hospitals. The survey included the Ward Nurses’ Discharge-Oriented Dietary Support Scale for Older Adult Patients (NDODSS), items assessing nurses’ perceived difficulty with cancer care, patient assessment, interprofessional collaboration, and their interest in, perceived importance of, and difficulty with dietary support. Data were analyzed using *t*-test, Pearson correlation, and multiple regression analysis. **Results**: Overall, 134 nurses, with an average of 6.8 years of cancer nursing experience, were included. The total mean scores for NDODSS, assessment of healthy eating behavior, adjustment of the living environment, and continual frailty assessment were 68.6 (11.8), 28.4 (4.9), 18.2 (4.7), and 22.0 (4.2), respectively. Multiple regression analysis showed that NDODSS was significantly associated with difficulty providing dietary support (β = −0.127, *p* = 0.043), physical assessment of cancer patients (β = 0.282, *p* < 0.001), social assessment (β = 0.207, *p* = 0.003), and consultation with other professionals (β = 0.205, *p* = 0.010). **Conclusions**: Dietary support for older patients with cancer requires a multidisciplinary approach, including cancer symptom assessment, social factor evaluation and sharing dietary support-related challenges.

## 1. Introduction

### 1.1. The Importance of Dietary Support for Older Patients with Cancer

In most countries, the proportion of people aged over 60 years is growing faster than that of people in other age groups [1]. The number of older patients with cancer is also increasing [2]. In Japan, in 2019, 75.5% of cancer cases occurred in patients over 65 years old, and in 2022, 88.3% of cancer deaths occurred in this population [3]. Patients with cancer are particularly at high risk of malnutrition, as tumor-derived inflammatory cytokines disrupt systemic carbohydrate, fat, and protein metabolism [4]. Malnutrition is associated with adverse postoperative outcomes (mortality, length of hospital stay, costs, and postoperative complications), increased toxicity, and treatment delays and is an independent predictor of survival [5,6]. In fact, the European Society for Clinical Nutrition and Metabolism guidelines for patients with cancer recommend nutritional support for patients with moderate malnutrition or those at risk of it [7]. Additionally, older adults are predisposed to malnutrition due to a combination of physical factors, psychological factors such as cognitive decline, and social factors, including living alone, eating alone, and limited food access [8,9,10]. Therefore, for older patients with cancer, a timely nutritional assessment and coordination of nutritional support must be planned to prevent nutritional status deterioration. Notably, artificial intelligence has been introduced in recent nutritional management for patients with cancer, enabling the identification of patients at risk who require nutritional care and the proposal of intervention programs [11]. These advances are expected to result in new approaches in nutritional management for patients with cancer.

### 1.2. Challenges in Dietary Support for Older Patients with Cancer

In Japan, medical service fees have promoted nutritional management by the nutrition support team (NST) [12]; nonetheless, because approximately 70% of inpatients at acute care hospitals are older adults and most NST members are not exclusively assigned to nutritional support, timely nutritional assessment and comprehensive nutritional support plans for all patients are difficult to manage. Therefore, it is important that registered nurses working in wards, who understand the daily living environments of patients, provide dietary support and further promote it in the future [13]. However, owing to short hospital stays and demanding work schedules, nurses often prioritize medical diagnosis and treatment of patients over nutritional care during hospitalization [14]. Moreover, a few studies have revealed that nurses face individual barriers in nutritional care, such as low confidence in providing this information or assessing nutritional status, limited awareness or use of guidelines [15,16], and insufficient knowledge, skills, and educational opportunities [17]. Nonetheless, these findings mainly concern general patients or older adults; research focusing on older patients with cancer is lacking. To enhance the practice of ward nurses in dietary support for older patients with cancer, it is first necessary to clarify the actual practice of such support by ward nurses and identify the related factors.

This study primarily aimed to clarify the actual dietary support practices for older patients with cancer by ward nurses at regional core cancer hospitals in Japan, while the secondary objective was to identify related factors of the actual practice of dietary support.

## 2. Materials and Methods

### 2.1. Study Design

We conducted a cross-sectional study using a self-report questionnaire. The sampling method was convenience sampling. The directors of the nursing departments at two regional core cancer hospitals in Japan were contacted to explain the research objectives and request their cooperation in distributing the questionnaire. Questionnaires were distributed via paper or web-based formats, and completed responses were collected from the wards.

### 2.2. Setting

This study was conducted from September 2024 to February 2025 at two regional core cancer hospitals in Sapporo, Hokkaido, Japan.

### 2.3. Participants

We targeted all registered nurses working in surgical and medical wards who routinely care for older patients with cancer. Registered nurses working in the wards were referred to as ward nurses.

The inclusion criterion was employment as a ward nurse with at least 1 year of work experience at a regional core cancer hospital in Japan. Ward managers and nurses with less than 1 year of nursing experience were excluded.

### 2.4. Variables

This self-administered questionnaire was developed and reviewed by the research team. Based on previous studies, variables were defined to assess the current state of dietary support for older cancer patients, nurse-related and organizational factors associated with such support, and teamwork essential for nutritional management. The questionnaire included items in the following key areas: (1) Ward Nurses’ Discharge-Oriented Dietary Support Scale for Older Adult Patients (NDODSS), (2) awareness and perceptions regarding dietary support for older patients with cancer, including perceived interest, importance, and difficulty, and (3) facilitating factors related to nurses’ practices for older patients with cancer. Furthermore, because this study focused on examining nursing practices related to dietary support for older patients with cancer, variables linked to knowledge and skills in cancer nursing—identified as facilitating factors for cancer nursing practice in previous studies—were also included [18,19]. In addition, the questionnaire collected data on the characteristics of registered nurses working in wards.

#### 2.4.1. Study Participant Characteristics

The questionnaire included questions on general characteristics of registered nurses working in wards, such as age, sex, years of nursing experience, years of cancer nursing experience, work experience on NST, participation in nutrition care training, and self-learning regarding nutrition management.

#### 2.4.2. Awareness and Perceptions Regarding Nutritional Management of Older Patients with Cancer

We assessed the levels of interest, importance, and difficulty regarding dietary support for older patients with cancer. Responses were provided on a 10-point scale, with scores ranging from 1 to 10 and higher scores indicating greater perceptions of interest, importance, and difficulty.

#### 2.4.3. NDODSS for Older Adult Patients

Participants were asked to assess their dietary support practices for older patients with cancer using the NDODSS developed by Miyabe [20]. Dietary support for older patients requires a comprehensive assessment of physical, psychological, and social background factors to prepare them for life after discharge from the hospital, and this scale includes these aspects. It comprises three subscales: (1) assessment of healthy eating behavior (8 statements); (2) adjustment of the living environment, with the involvement of family members and caregivers, as well as other professionals (6 statements); and (3) continual frailty assessment (6 statements). The scale comprises 20 statements, with responses rated on a five-point Likert scale (1 = never, 2 = rarely, 3 = neutral, 4 = often, and 5 = always). The total scores range from 20 to 100, with higher scores indicating more nursing practice. The Cronbach’s alpha coefficients were 0.932 for the overall scale, 0.887 for Factor 1, 0.885 for Factor 2, and 0.862 for Factor 3. This is a validated instrument with confirmed reliability and validity.

#### 2.4.4. Knowledge About Cancer Nursing

Participants were requested to evaluate their knowledge about cancer nursing using the Nurses’ Difficulty with Cancer Care (NDCC) [21]. For the present survey, only the “knowledge and skills” subscale was used. This subscale allows nurses to self-assess their knowledge and skills for essential cancer nursing, including radiation therapy, drug therapy, surgical therapy, and symptom management associated with these treatments. It comprises nine statements, with responses rated on a six-point Likert scale (1 = strongly disagree, 2 = disagree, 3 = slightly disagree, 4 = slightly agree, 5 = agree, and 6 = strongly agree). The total scores range from 9 to 54, with lower scores indicating greater knowledge about cancer nursing. Cronbach’s alpha coefficient was 0.73 for this subscale. This is a validated instrument with confirmed reliability and validity.

#### 2.4.5. Contributing Factors Related to Nurses’ Practice for Older Patients with Cancer

We assessed contributing factors related to nurses’ practice for older patients with cancer through the following questions: (1) Do you assess cancer-specific symptoms and conduct a social assessment of cancer patients? This comprises six statements addressing the influence of fatigue, dyspnea, insomnia, eating alone, and food access on nutritional status, with responses rated on a five-point Likert scale. A principal component analysis was conducted to confirm that these items had a one-factor structure. (2) Do you conduct nutritional assessment screening during hospitalization and use tools to assess patients’ nutritional status? (3) Do you share information with colleagues regarding patient nutrition management? (4) Do you consult multiple healthcare professionals regarding the nutritional management of patients? (5) Do you collaborate with outpatient clinics regarding the nutritional management of patients? (6) Do you provide information to patients and their families regarding social resources for nutritional management? Responses were rated on a five-point Likert scale (1 = never, 2 = rarely, 3 = neutral, 4 = often, and 5 = always). The scores range from 1 to 5, with higher scores indicating greater nursing practice.

### 2.5. Sample Size

We calculated the sample size using G*Power 3.1.9.6 [22]. Based on 10 predicted independent variables for multiple regression analysis, with a medium effect size of 0.15, a significance level of 5%, and a power of 80%, the required sample size was calculated to be 118 cases. We estimated an approximate 40% response rate and needed to distribute 295 questionnaires.

### 2.6. Data Analysis

All the statistical analyses were performed using SPSS, version 26.0 (IBM Corp., Armonk, NY, USA). Based on a previous study [20], in the NDODSS, missing values for three or fewer items were replaced with the mean values of the other items. Descriptive statistics were used to characterize registered nurses working inwards, along with their NDODSS scores, knowledge of cancer nursing, and perceived interest, importance, and difficulty regarding dietary support for older patients with cancer (i.e., mean with standard deviation [SD] and percentage). We evaluated each scale’s reliability using Cronbach’s α. We hypothesized that dietary support for older patients with cancer would be associated with “Characteristics of study participants,” “Awareness and perceptions regarding nutritional management of older patients with cancer,” “Knowledge about cancer nursing,” and “Collaboration.” Pearson’s product-moment correlation analysis and a two-sample *t*-test were used to analyze the relationships between NDODSS and other variables. All significant variables (*p* < 0.05) from the univariate analysis were included as independent variables in the forced entry method to identify the main factors influencing NDODSS as the dependent variable. The forced entry method in multiple regression is a technique where all selected independent variables are entered into the model simultaneously rather than stepwise. This approach treats all predictors equally and is often based on theoretical considerations [23]. Variables with high multicollinearity were excluded from multiple regression analysis. Statistical significance was set at *p* < 0.05.

### 2.7. Ethical Considerations

This survey was approved by the Ethics Committee of the Graduate School of Health Sciences, Hokkaido University, on 5 August 2024 (No. 24-43). Informed consent was obtained from all participants, with instructions specifying that returning the questionnaire was considered consent. The informed consent emphasized voluntary participation, anonymity, and confidentiality.

## 3. Results

### 3.1. Study Population

A total of 304 questionnaires were distributed, and 136 were returned (response rate: 44.7%). Two questionnaires were excluded due to missing responses on the NDODSS. Therefore, 134 responses were analyzed (valid response rate: 44.1%).

### 3.2. Study Participant Characteristics

The age distribution was as follows: 76 participants were in their twenties (56.7%), 37 in their thirties (27.6%), 17 in their forties (12.7%), and four in their fifties (3.0%). The mean (SD) durations of nursing experience and cancer nursing experience were 8.8 (7.2) and 6.8 (5.6) years, respectively (Table 1). Eleven (8.2%) participants had worked on an NST; 14 (10.4%) had participated in nutrition care training; 59 (44.0%) had reflected on nursing practice related to nutrition management; and 51 (38.1%) had learned about nutrition management.

### 3.3. Awareness and Perceptions Regarding Nutritional Management of Older Patients with Cancer

The mean (SD) scores for perceived interest, importance, and difficulty related to dietary support for older patients were 6.8 (1.7), 8.0 (1.6), and 5.6 (1.8), respectively. A total of 41 participants (30.6%) used tools to assess nutritional status.

### 3.4. Ward Nurses’ Discharge-Oriented Dietary Support for Older Adult Patients

The Cronbach’s α on NDODSS was 0.90 for the overall scale items and ranged from 0.77 to 0.81 for the subscales, confirming its internal consistency. The mean (SD) scores for the NDODSS, assessment for healthy eating behavior, adjustment of the living environment, and continual frailty assessment were 68.6 (11.8), 28.4 (4.9), 18.2 (4.7), and 22.0 (4.2), respectively (Table 2). The item average scores of the subscales were 3.6, 3.0, and 3.7, respectively.

### 3.5. Knowledge About Cancer Nursing

The Cronbach’s α on NDCC was 0.91 for the “knowledge and skills” subscale, confirming its internal consistency. The mean (SD) score for knowledge about cancer nursing was 31.7 (7.3) (Table 2).

### 3.6. Factors Related to the NDODSS for Older Adult Patients

Single regression analysis showed no valid association between NDODSS and years of nursing experience, years of cancer nursing experience, NST work experience, or participation in nutrition care training. Nurses who reflected on their nutrition management practice had a significantly higher mean score than those who did not (*p* < 0.001). Similarly, nurses who received education on nutrition management had a significantly higher mean score compared to those who did not (*p* = 0.019) (Table 3). Regarding knowledge and perception about cancer nursing, the following variables showed correlations: knowledge about cancer nursing (*p* = 0.01), perceived interest (*p* < 0.01), and perceived difficulty (*p* < 0.01); nevertheless, no valid association was observed for perceived importance. The associations between NDODSS and contributing factors related to nurses’ practice for older patients with cancer were as follows: assessment of cancer-specific symptoms (*p* < 0.01), social assessment of patients with cancer (*p* < 0.01), sharing information with colleagues regarding nutrition management (*p* < 0.01), consulting with multiple healthcare professionals regarding nutritional management (*p* < 0.01), collaborating with outpatient clinics regarding nutrition management (*p* < 0.01), and providing information to patients and families regarding social resources for nutritional management (*p* < 0.01). Nonetheless, no significant associations were observed between the implementation of nutritional assessment screening during hospitalization and the use of tools to assess patients’ nutritional status (Table 4).

No high collinearity was observed across variables. The scale was used as the dependent variable, and the following items, for which a statistically significant difference was found at the 5% level, were used as the independent variables: reflecting on nursing practice related to nutrition management, learning about nutrition management, knowledge about cancer nursing (nurses’ difficulty with cancer care), perceived interest regarding dietary support for older patients with cancer, perceived difficulty regarding dietary support for older patients with cancer, assessment of cancer-specific symptoms, social assessment of cancer patients, sharing information with colleagues, consulting with multiple healthcare professionals, collaborating with outpatient clinics, and providing information to patients and families regarding social resources for nutritional management.

Multiple regression analysis showed a significant association between NDODSS and perceived difficulty regarding dietary support for older patients with cancer (β = −0.127, *p* = 0.043), assessment of cancer-specific symptoms (β = 0.282, *p* < 0.001), social assessment of patients with cancer (β = 0.207, *p* = 0.003), and consulting with multiple healthcare professionals regarding nutritional management (β = 0.205, *p* = 0.010; R^2^ = 0.597, adjusted R^2^ = 0.560, *p* < 0.001) (Table 5).

## 4. Discussion

This study revealed the actual practice of dietary support for older patients with cancer by ward nurses at regional core cancer hospitals in Japan and its associated factors.

Compared with a previous study [20], the total and subscale scores of dietary support practices for older adults tended to be slightly higher in our study. Nonetheless, the score for the subscale “adjustment of the living environment” was lower than that of the other subscales. Older patients with cancer have greater physical impairments and need to adjust their living environments after discharge from the hospital [24]. Nevertheless, previous research has identified a lack of time to complete the report and poor information about nursing diagnoses and patients’ social assessment as problematic areas [25]. Here, similar results are presumed to have been observed in dietary support as well. Specifically, this subscale includes items related to dietary rehabilitation, utilization of home-based resources, and living environment adaptation. Because matters involving this multidisciplinary collaboration are often handled not by ward nurses themselves but by the Community Liaison Department or NST as needed, the score may appear low in the survey results. Conversely, scores for the subscales “assessment for healthy eating behavior” and “continual frailty assessment” were high. In acute care hospitals, owing to short hospital stays and demanding work schedules, nurses often prioritize medical diagnosis and treatment over nutritional care during hospitalization [14]. Therefore, these subscales related to treatment and cancer disease were considered relatively well implemented.

Second, the factors associated with dietary support practice in this study are discussed as follows. Among individual nurse factors, knowledge about cancer nursing and the perceived difficulty level of dietary support for older patients with cancer were identified as barriers to dietary support, whereas learning about nutrition management was identified as a facilitating factor. This likely contributes to the perceived difficulty of dietary support, stemming from low confidence in providing this information or assessing nutritional status and from limited awareness or use of guidelines [15,16]. Lack of nutrition education among nurses and the burden of multiple responsibilities beyond dietary care have been identified as barriers to effective support [26]. This result emphasizes the importance of educational opportunities for nurses to improve awareness of dietary support, consistent with previous research showing that improved knowledge leads to positive perceptions and further improves practice [27]. Additionally, because reflection on nursing practice related to nutrition management is a dietary support facilitating factor, establishing regular nursing practice evaluation through ward conferences is effective. Furthermore, given the limited awareness or use of guidelines [15,16], it is necessary to establish a practical ward-level implementation system, such as more actively promoting guideline adoption.

The assessment of nutrition-related effects of cancer-specific symptoms had the strongest influence on the implementation of dietary support in older patients with cancer. With an increasing number of older adults undergoing cancer treatment, the risk of malnutrition grows due to aging, cancer-related inflammatory and metabolic abnormalities [4], and treatment-related side effects [28]. Symptoms arising from cancer or its treatment can serve as barriers to adequate nutritional intake [29]. Enhancing nurses’ understanding of how symptom assessments during cancer treatment affect nutrition may promote dietary support practices and improve patients’ quality of life and prognosis. This requires using measures such as symptom assessment tools and providing educational opportunities to improve knowledge of symptoms and nutrition management. The second most influential factor was social assessment, such as the evaluation of patients’ living arrangements. In particular, psychosocial factors have been suggested to exert a significant impact on the nutritional status of older adults [30,31,32]. However, it is often difficult for nurses to obtain detailed information about patients’ social backgrounds during a short hospital stay. In fact, it would be better if nurses could conduct assessments with other related professionals rather than working only with nurses. The third factor associated with dietary support practice was interprofessional collaboration, highlighting the importance of a multidisciplinary approach to nutritional management. The result supports previous studies on the need to promote interprofessional practice in the nutrition care of patients with cancer [33]. Japan’s 2024 revision of the medical fee schedule [12] introduced a new reimbursement category for “Multidisciplinary Collaboration in Nutritional Management.” In particular, older patients often live alone or in a couple of households, which requires coordination of various resources. Therefore, effective nutritional support requires collaboration among physicians, nurses, registered dietitians, pharmacists, and rehabilitation staff, with sharing of information gathered from multiple perspectives, thereby enabling dietary support that better considers the individuality of each patient. The fourth factor, the difficulty level of dietary support for older patients with cancer, was identified as a key influential variable among the related factors. To address barriers associated with this perceived difficulty, a multidisciplinary team approach is essential, and educational opportunities that empower nurses to deliver dietary care with confidence and competence are crucial.

This study suggests that, to improve dietary support practices for older patients with cancer, both individual factors—such as enhancing nurses’ knowledge and symptom assessment practices —and organizational initiatives are necessary to establish an effective multidisciplinary collaboration system. For future implementation of more effective dietary support, previous studies have suggested the potential effectiveness of automated nutrition screening and monitoring systems utilizing electronic medical records [34]. Therefore, rather than relying solely on the individual efforts of ward nurses or the specific activities of the NST, integrating digital health technologies (medical DX) into dietary support for patients undergoing cancer treatment should be considered an important future direction.

## 5. Limitations

This study has some limitations. First, it was conducted in a limited number of regional core cancer hospitals in Japan, which may affect generalizability. Second, response bias may have been introduced if participants were mainly those interested in nutritional support. To clarify barriers and facilitating factors associated with dietary support for older patients with cancer, a larger sample size and qualitative research to clarify the nature of difficulties experienced by nurses are required.

## 6. Conclusions

In providing discharge-oriented dietary support for older patients with cancer, support related to adjusting the living environment may be insufficient. Symptom assessment, social environment assessment, and interprofessional collaboration were identified as facilitators of dietary support practices, whereas perceived difficulty in providing such support was identified as a barrier. Therefore, enhancing nurse assessment skills by providing educational and reflective opportunities and implementing organizational initiatives such as establishing collaborative systems and integrating digital health technologies is necessary in dietary support for older patients undergoing cancer treatment.

## Figures and Tables

**Table 1:** Study Participant Characteristics.

		Mean (SD)	N	%	Min	Max
Age	Twenties		76	56.7		
	Thirties		37	27.6		
	Forties		17	12.7		
	Fifties		4	3.0		
Sex	Female		120	89.6		
	Male		14	10.4		
Years of nursing experience		8.8 (7.2)	133		2	37
Years of cancer nursing experience		6.8 (5.6)	132		0	30

Abbreviations: SD, standard deviation; Min, minimum; Max, maximum.

**Table 2:** The Ward Nurses’ Discharge-Oriented Dietary Support Scale for Older Adult Patients, Knowledge about Cancer Nursing.

	Range (Items)	Mean (SD)	Item Average	Min–Max
Subscale 1. Assessment for healthy eating behavior	8–40 (8)	28.4 (4.9)	3.6	11–40
Subscale 2. Adjustment of the living environment, with the involvement of family members and caregivers, along with other professionals	6–30 (6)	18.2 (4.7)	3.0	6–28
Subscale 3. Continual frailty assessment	6–30 (6)	22.0 (4.2)	3.7	10–30
Total score of the Ward Nurses’ Discharge-Oriented Dietary Support Scales for Older Adult Patients	20–100 (20)	68.6 (11.8)		31–94
Knowledge about cancer nursing (NDCC)	9–54 (9)	31.7 (7.3)		9–54

Note: Knowledge About Cancer Nursing (NDCC): The “Knowledge and Skills” subscale consists of nine statements that ask nurses to self-evaluate their knowledge of essential aspects of cancer treatment, including drug therapy, surgical therapy, radiation therapy, and symptom management. Abbreviations: SD, standard deviation; Min, minimum; Max, maximum.

**Table 3:** Differences in NDODSS Scores according to Nurse-Related Factors.

		N	Mean	SD	*p*
Work experience on an NST	yes	11	71.4	13.0	0.415
	no	123	68.3	11.7	
Participation in nutrition care training	yes	14	68.3	11.6	0.884
	no	119	68.8	11.8	
Reflecting on nursing practice related to nutrition management	yes	59	72.7	10.4	0.000
	no	74	65.3	12.0	
Learning about nutrition management	yes	51	71.6	11.5	0.019
	no	82	66.7	11.7	
Using tools to assess patients’ nutritional status	yes	41	71.0	10.8	0.103
	no	81	67.5	11.5	

Two-sample *t*-test. Abbreviations: SD, standard deviation; NST, nutrition support team.

**Table 4:** Correlation between Nurse-Related Factors and NDODSS Scores.

	*r*	*p*
Years of nursing experience	−0.125	0.152
Years of cancer nursing experience	−0.094	0.286
Knowledge about cancer nursing (nurses’ difficulty with cancer care)	−0.223	0.010
Interest level of dietary support for older patients with cancer	0.253	0.003
Importance level of dietary support for older patients with cancer	0.058	0.509
Difficulty level of dietary support for older patients with cancer	−0.274	0.001
Assessment of cancer-specific symptoms	0.535	0.000
Social assessment of cancer patients	0.467	0.000
Conducting nutritional assessment screening during hospitalization	0.117	0.177
Sharing information with colleagues regarding the nutritional management of patients	0.497	0.000
Consulting with multiple healthcare professionals regarding the nutritional management of patients	0.513	0.000
Collaborating with outpatient clinics regarding the nutritional management of patients	0.396	0.000
Providing information to patients and families regarding social resources for nutritional management	0.417	0.000

Pearson’s product-moment correlation analysis. Abbreviations: r, correlation coefficient.

**Table 5:** Multiple Regression Analysis: Factors Related to the NDODSS for Older Adult Patients.

	Total Score				
				95% CI of B	
	*β*	*t*	*p*	Lower	Upper
Reflect on nursing practice related to nutrition management	0.037	0.481	0.632	−2.754	4.519
Learning about nutrition management	0.060	0.810	0.420	−2.109	5.030
Knowledge about cancer nursing (nurses’ difficulty with cancer care)	−0.102	−1.648	0.102	−0.362	0.033
Interest level of dietary support for older patients with cancer	0.092	1.430	0.155	−0.240	1.488
Difficulty level of dietary support for older patients with cancer	−0.127	−2.042	0.043	−1.626	−0.025
Assessment of cancer-specific symptoms	0.282	4.271	0.000	0.609	1.661
Social assessment of cancer patients	0.207	3.052	0.003	0.427	2.005
Sharing information with colleagues regarding the nutritional management of patients	0.117	1.441	0.152	−0.516	3.276
Consultation with multiple healthcare professionals regarding the nutritional management of patients	0.205	2.633	0.010	0.646	4.561
Collaborate with outpatient clinics regarding the nutritional management of patients	0.074	1.088	0.279	−0.636	2.185
Provides information to patients and families regarding social resources for nutritional management	0.124	1.864	0.065	−0.079	2.633
R^2^	0.597				
Adj R^2^	0.560				
F for change in adj R^2^	16.172	(*p* < 0.001)			

Note: Assessment of cancer-specific symptoms: influence of fatigue, dyspnea, and insomnia. Social assessment of cancer patients: effect of eating alone or food access on nutritional status. Multiple regression analysis was performed using the forced entry method. Abbreviations: β, standard partial regression coefficient; B, non-standardization factor; CI, confidence interval; R^2^, coefficient of determination.

## Data Availability

All data are included within the article, and additional data are available from the corresponding author upon reasonable request. The data are not publicly available due to privacy considerations.

## Public Involvement Statement

No public involvement in any aspect of this research.

## Guidelines and Standards Statement

This manuscript was prepared in accordance with the STROBE (Strengthening the Reporting of Observational Studies in Epidemiology) statement, which provides guidelines for the reporting of observational studies [35].

## Abbreviations

The following abbreviations are used in this manuscript:

CI, confidence interval; NDCC, Nurses’ Difficulty with Cancer Care; NDODSS, Ward Nurses’ Discharge-Oriented Dietary Support Scale for Older Adult Patients; NST, nutrition support team; SD, standard deviation.
